# Exploring wireless device-free localization technique to assist home-based neuro-rehabilitation

**DOI:** 10.3389/fnins.2024.1344841

**Published:** 2024-02-02

**Authors:** Zhen Wang, Xiaoou Li, Guoli Wang

**Affiliations:** ^1^College of Medical Instrumentation, Shanghai University of Medicine & Health Sciences, Shanghai, China; ^2^School of Computer Science and Engineering, Sun Yat-sen University, Guangzhou, China

**Keywords:** rehabilitation, localization, RTI, RSS, fluctuation-level

## Abstract

Home-based movement neuro-rehabilitation is quite necessary when the patient goes back home from hospital. Due to lack of supervision from doctors, rehabilitation at home is often forgotten. As an alternate to doctor-supervision, in this research, we explore the wireless device-free localization technique to assist the rehabilitation procedure. The localization technique can judge whether the patient is near the rehabilitation equipment and even obtain the movement trajectory. The most challenging problem in the wireless device-free localization system is that the received-signal-strength (RSS) of the electromagnetic-wave is unpredictable, which increases the localization error. How to select the informative RSS is pretty important. This research proposes a new criterion (i.e., fluctuation-level) to select the informative RSS. Experimental results show the effectiveness of the proposed fluctuation-level in reducing the localization error.

## 1 Introduction

For the patient who suffers from neuro-disabilities, he/she need to perform neuro-rehabilitation under the supervision of rehabilitation doctors. Usually, after he leaves the hospital, he is still required to do daily rehabilitation at home by himself (Maresca et al., 2020). However, due to lack of supervision from the rehabilitation doctors, the patient often forgets to take rehabilitation-exercises, leading to the degradation in health condition. To tackle this problem, technology-assisted movement-evaluation can be adopted (Zhang et al., 2020; Hu et al., 2023). One fundamental technique is to obtain the indoor location of the patient, which can judge whether the patient is near the rehabilitation equipment.

Currently, the indoor localization technique mainly adopts device-based strategy, meaning that the target needs to attach a device to obtain his location (Cao et al., 2020). Device-based strategy is inappropriate for the patient localization because the device hinders the patient to move and take rehabilitation-exercises. Therefore, this research explores the device-free localization technique to supervise the patient to take home-based rehabilitation.

Technically, many sensing strategies can be employed to achieve device-free localization, such as camera-based vision localization (Kim and Jun, 2008), infrared-based localization (Ngamakeur et al., 2022), ultrasound localization (Yoon and Park, 2016), RF (radio frequency) based wireless localization (Khan et al., 2021; Abdullah et al., 2023) etc. Camera-based localization somehow demands huge memory for video storage. Infrared-based localization is fragile to be interfered by fluorescent light, and the localization distance is limited. Ultrasound-based localization is easily influenced by Doppler effect and the localization area is comparably small. By contrast, RF-based localization has the advantages of traveling long distance, not influenced by light, low data-storage, not violating the privacy etc., which attracts much more attention in the research community.

RF-based localization employs the principle that the wireless signal would change in received-signal-strength (RSS), channel state information (CSI), phase of arrival (POA) or angle of arrival (AOA) after the presence of the target. In which, RSS is the widely used pattern. Among different methods in RSS-based localization, radio-tomographic imaging (RTI) is an effective yet simple approach (Wilson and Patwari, 2010; Zhen et al., 2019a) which is the focus of this article.

RTI considers the located area as one shadowing image, each pixel has its pixel-value. The pixel-value equals to the attenuation-value occurring at the location of this pixel. RTI first computes one shadowing image from the RSS measurements. Then, the patient's location is estimated by finding the position with maximum pixel-value in the image (Zhen et al., 2019b).

However, there is one challenge which degrades the localization performance. The challenge is that not all the RSS measurements are contributive for patient-localization due to the multipath-noise in wireless propagation. How to select the informative RSS measurements that contribute to indoor-localization is the essential problem in RTI. Aiming at addressing this problem, current literatures mainly adopt the criterion of fade-level to select the informative RSS measurements (Wilson, 2012; Kaltiokallio et al., 2013; Mela et al., 2023). However, in practice, only using fade-level to select informative RSS measurements is unsatisfactory. In this article, in addition to fade-level, we propose another criterion (i.e., fluctuation-level) to select the informative RSS measurements. Experimental results show that combining fade-level and the proposed fluctuation-level can achieve better localization performance than just using fade-level.

## 2 Method

### 2.1 Wireless network deployment

**Figure 1:**
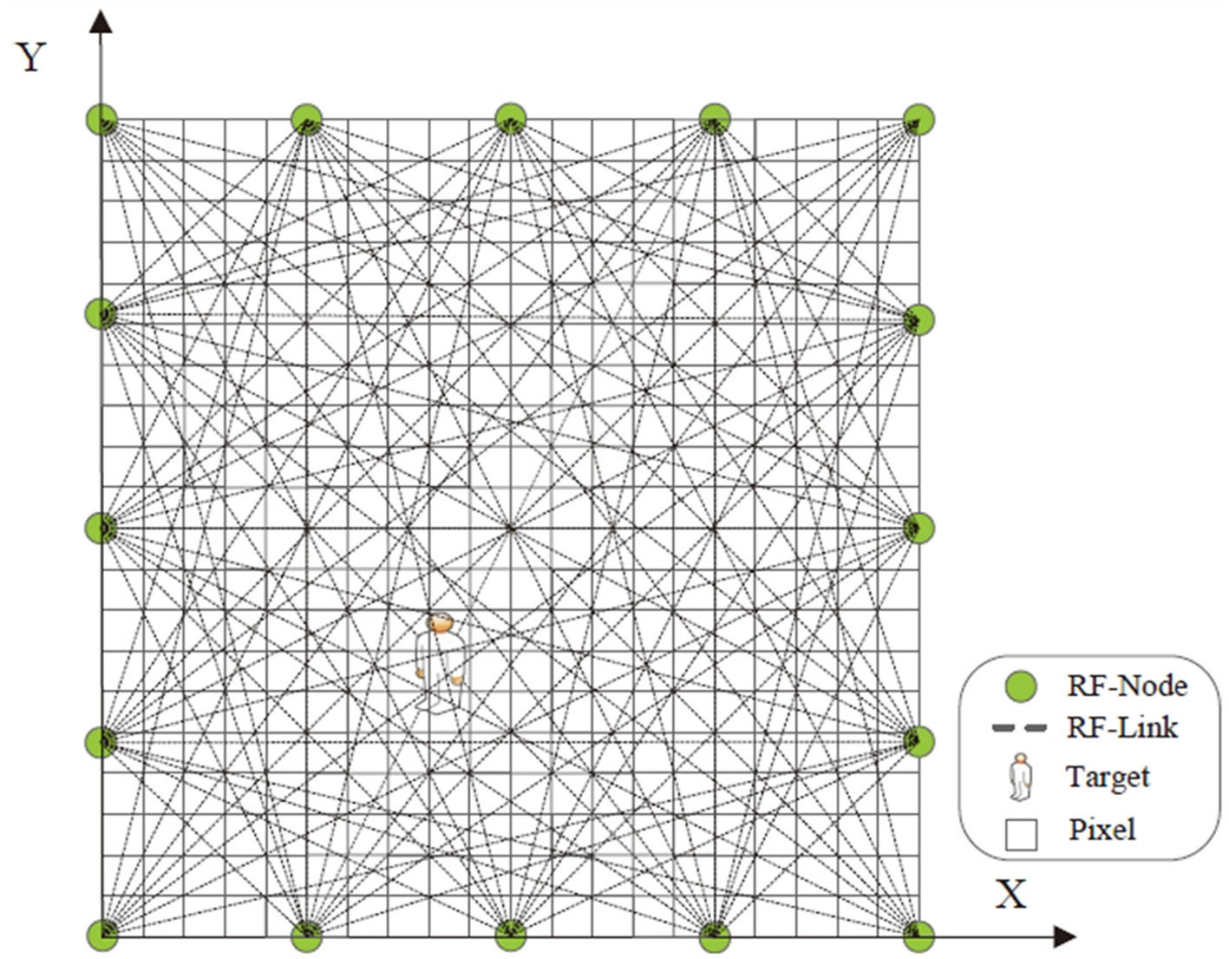
Wireless network deployment. Green circle represents the RF node. Dashed line denotes the RF link between two node-pairs. The target is within the located area covered by the wireless network. Square represents the pixel in the shadowing image.

Before implementing the device-free localization system, a wireless network should be deployed to cover the indoor area. Figure 1 illustrates one example of the wireless network. Each RF node adopts the off-the-shelf circuit-board which can guarantee the low system cost of the localization system. Each node-pair constitutes one RF-link. The area is divided into several pixel-grids, and each pixel has its coordinate. All the pixels constitute one shadowing image. The pixel-value denotes the attenuation-value occurring at the coordinate of this pixel. Intuitively, the pixel where the patient locates has maximum attenuation-value (or pixel-value). Therefore, we can obtain the location of the patient according to the pixel-value of the shadowing image. More RF nodes can contribute to enhance the localization accuracy. However, the trade-off should be made between localization accuracy and increased system cost in engineering practice.

In mathematics Equation (1), can be used to model RTI (Wilson and Patwari, 2010),


(1) y=Hx+n,

where $y={\left[y_{1},\cdots \;,y_{M}\right]}^{T}$ represents the RSS measurements (i.e., the RSS variation before and after the patient presents in the room), *M* equals to the total number of RF-links in the wireless network. $x={\left[x_{1},\cdots \;,x_{N}\right]}^{T}$ is the image-vector with each element denoting the attenuation-value, *N* is the pixel number. $n={\left[n_{1},\cdots \;,n_{M}\right]}^{T}$ is the noise vector. ***H*** ∈ ℝ^*M*×*N*^ is the weighting matrix, which can be determined by the ellipse model (Wilson and Patwari, 2010).

The aim of RTI is to compute the image-vector *x* given the measured RSS *y*. Because not all the RSS is contributive for target localization, how to select the informative RSS constitutes one key problem in RTI-based device-free localization.

### 2.2 Selection of informative RSS by using fluctuation-level and fade-level

Currently, fade-level is the only criterion to evaluate whether the RSS from a particular link is informative or not. The computation of fade-level is shown as Equation (2),


(2) $$F_{l}={\bar{z}}_{l}-P(d_{l})$$

where *F*_*l*_ represents fade-level of the *l-th* link, $\bar{z}$ is the average RSS of the *l-th* link when the room is without any person, *P*(*d*_*l*_) denotes the RSS computed by the path-loss model (Wilson, 2012). Previous research shows that the RSS with large-positive fade-level is more informative for localization (Wilson, 2012).

However, fade-level just reflects the deviation property of the RSS. In practice, the localization accuracy when just using fade-level to select informative RSS is usually unsatisfactory. Here we explore the fluctuation property of the RSS, and take it as a new criterion for informative RSS selection. We name the fluctuation property as fluctuation-level. The fluctuation-level can be obtained by computing the RSS variance. Mathematically, the fluctuation-level is shown as Equation (3),


(3) $$\begin{array}{l} v_{l}=\frac{1}{W}\;\overset{W}{\underset{i=1}{\sum }}{\left({\tilde{r}}_{i}-{\bar{r}}_{l}\right)}^{2} \end{array}$$

where *v*_*l*_ denotes the fluctuation-level of the *l-th* link, *W* is the window length. ${\tilde{r}}_{i}$ denotes each RSS sample in this window, ${\bar{r}}_{l}$ is the mean of the RSS samples in this window for the *l-th* link. Intuitively, the RSS with lower fluctuation-level are more reliable than that with higher fluctuation-level.

In the localization system, we jointly use fluctuation-level and fade-level to select the informative RSS. Specifically, the fade-level and fluctuation-level are allocated with their respective threshold values *F*_*thr*_ and *v*_*thr*_. In the joint-selection strategy, if *F* > *F*_*thr*_ and *v* < *v*_*thr*_ are satisfied for a particular link, the RSS from this link can be considered as informative. Otherwise, the RSS is filtered as being uninformative. Mathematically, this joint-selection strategy can be written as Equation (4),


(4) $$link\_indicator=\{\begin{array}{ll} 1 & if\;F>F_{thr}\;and\;v<v_{thr}\\ 0 & otherwise \end{array}$$

where the variable *link*_*indicator* is a binary variable {0, 1}, indicating whether the RSS from a particular RF-link is informative. Logic one indicates the link is informative, while zero denotes the contrary.

### 2.3 Shadowing image reconstruction and position inference

Based on the selected RSS, this section introduces how to compute the shadowing image and infer the patient's location. Because the image is sparse (Wilson and Patwari, 2010; Zhen et al., 2018), here the sparse Bayesian learning (SBL) method is adopted. In SBL, the image is allocated with zero-mean Gaussian prior-distribution having the variance constrained by inverse-Gamma distribution, which is expressed as Equation (5),


(5) $$\{\begin{array}{l} P(x|\lambda )=\prod_{i=1}^{N}Gauss(x_{i}|0,\lambda_{i})\\ P(\lambda_{i})=InvGamma(\lambda_{i}|a,b) \end{array}$$

where λ_*i*_ is the variance, *a, b* denote the shape and scale parameters respectively. For the noise, it is allocated with hierarchical prior-distribution shown as Equation (6),


(6) $$\{\begin{array}{l} P(n|\eta )=\prod_{i=1}^{M}Gauss(n_{i}|0,\eta_{i}^{-1})\\ P(\eta_{i})=Gamma(\eta_{i}|c,d) \end{array}$$

where $\eta_{i}^{-1}$ denotes the variance, *c, d* represent the shape and scale parameters in Gamma distribution. The conditional distribution is assigned with Gaussian distribution shown as Equation (7),


(7) $$\begin{array}{l} P\left(y|x,\eta \right)=N\left(y|Hx,D^{-1}\right) \end{array}$$

here ***D*** **=** *diag*(***η***), $\eta ={\left[\eta_{1},\eta_{2},\cdots \;,\eta_{M}\right]}^{T}$.

Based on probability theorem (Tipping, 2001; Ying et al., 2023), the posterior estimation can be obtained as Equation (8),


(8) $$\begin{array}{l} P(x|y;\lambda ,\eta )=\frac{P(y|x,\eta )P(x|\lambda )}{\int P(y|x;\eta )P(x|\lambda )dx}\\ \;=(\frac{1}{\sqrt{2\pi }}{)}^{M}|\Sigma {|}^{-\frac{1}{2}}\exp\left[-\frac{1}{2}{\left(x-\mu \right)}^{T}\Sigma^{-1}(x-\mu )\right] \end{array}$$

where ***μ*** is the posterior-mean of the shadowing image, **Σ** is the covariance. Their respective expressions are stated in Equations (9, 10).


(9) $$\mu =\Sigma H^{T}Dy$$

(10) $$\Sigma ={\left[H^{T}DH+C\right]}^{-1}$$

here ***C*** = *diag*(**λ**) with $\lambda ={\left[\lambda_{1},\lambda_{2},\cdots \;,\lambda_{M}\right]}^{T}$.

As to $\lambda ={\left[\lambda_{1},\lambda_{2},\cdots \;,\lambda_{N}\right]}^{T}$, $\eta ={\left[\eta_{1},\eta_{2},\cdots \;,\eta_{M}\right]}^{T}$, they can be expressed as Equation (11),


(11) $$\{\begin{array}{l} \lambda_{i}=\frac{2a+\theta_{i}-2}{2b+\mu_{i}^{2}}\\ \eta_{j}=\frac{2c-1}{2d+tr(\Sigma H_{j}^{T}H_{j})+{\left(y_{i}-H_{j}\mu \right)}^{2}} \end{array}$$

where *θ*_*i*_ = 1 − λ_*i*_Σ_*ii*_, in which Σ_*ii*_ stands for the *i-th* diagonal element in **Σ**. μ_*i*_ denotes the *i-th* number in ***μ***. ***H***_*j*_ is the *j-th* row of the weighting matrix ***H***. After the shadowing image is obtained, we can estimate the patient's location by finding the position where the maximum pixel-value locates. The localization algorithm is summarized in Algorithm 1.

**Algorithm 1:**
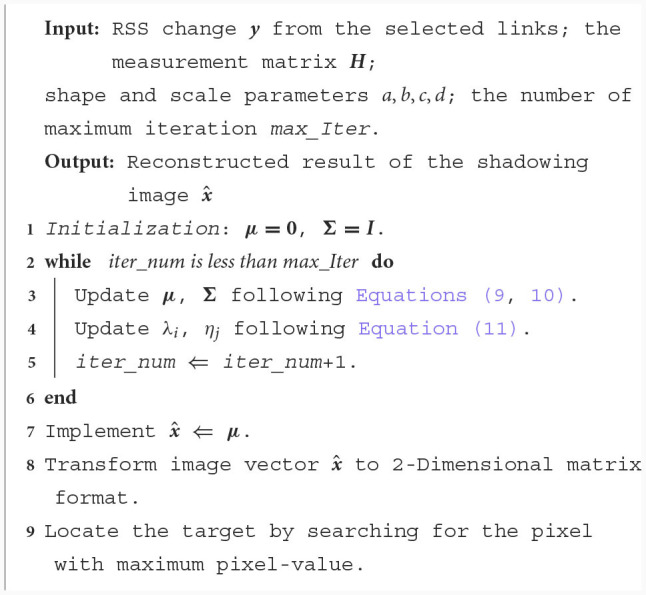
Localisation algorithm

## 3 Experiment and result

### 3.1 Experiment setup

**Figure 2:**
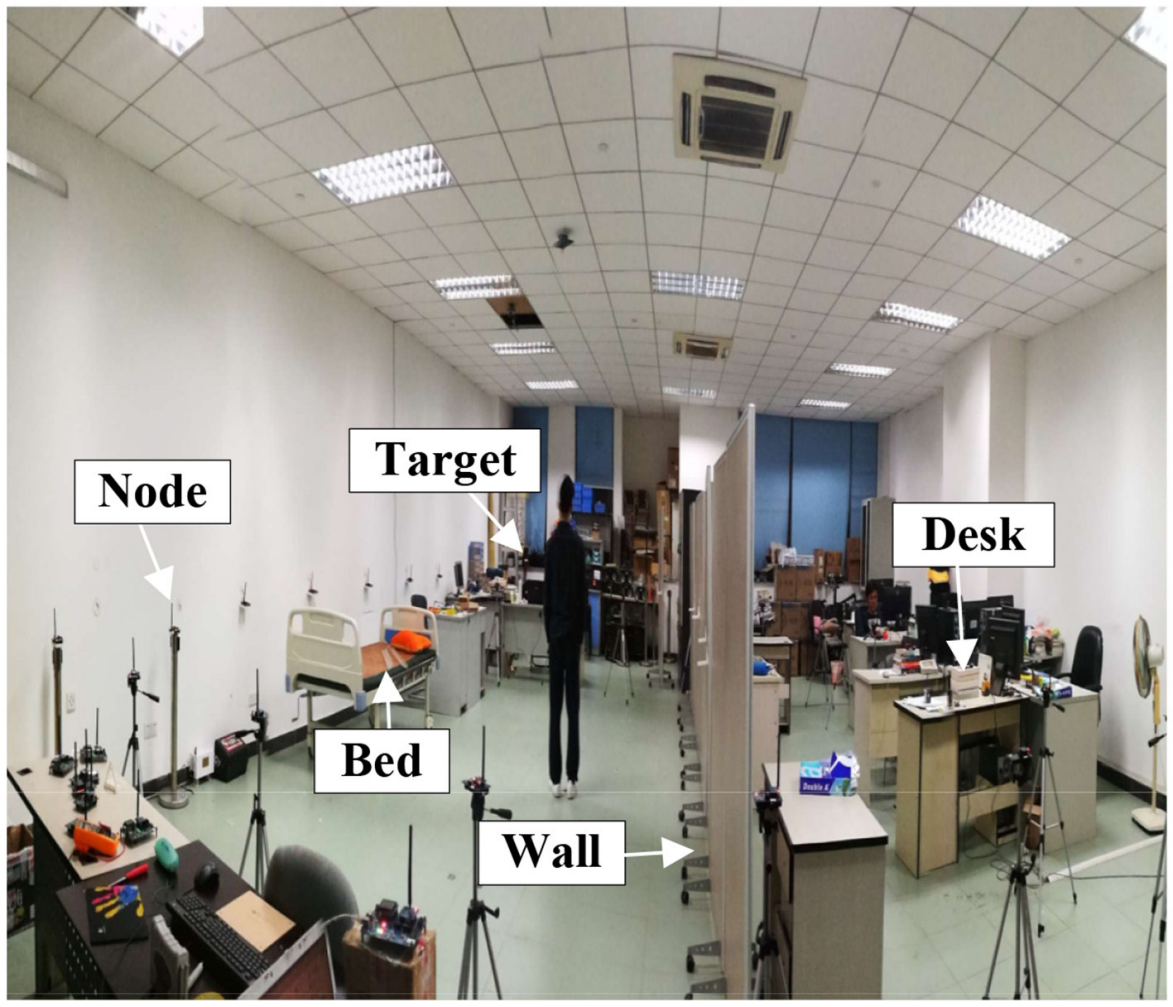
Experiment scenario. Lots of furniture are included in the room to constitute the environment with rich multipath propagation.

The localization experiment was carried out in an indoor 7.2 m * 7.5 m area (shown as Figure 2) with 24 nodes to constitute a wireless network. The RF nodes communicate following the ZigBee protocol with 2.4 GHz frequency-band. Every node has its node-identifier, which is used as the guidance of sequential transmission. At any instant, only one node transmit. Outside the wireless network, a special RF-node exists serving as the base-station. The base-station uploads the packet to the computer. The application program on the computer extracts the RSS from the received packet. In the experiment, the computer equips with Intel 2.4 GHz CPU and 4 GB RAM. In engineering practice, the localization algorithm can be run on the embedded system with low-cost.

### 3.2 Localization result

To evaluate the localization performance, the target sequentially stands at 31 different positions shown as Figure 2. For each position, all the packets are uploaded to the computer and the RSS are extracted. Then, two different RSS selection strategies (i.e., only fade-level v.s combining fluctuation-level and fade-level) are adopted to select the informative RSS. The selected RSS measurements by two strategies are respectively used for the computation of target's position following the procedure in Algorithm 1.

To quantitatively examine the localization performance, the localization error is used which is defined as Equation (12),


(12) $$e_{loc}=\|\hat{P}-P_{true}{\|}_{\ell_{2}}$$

where *e*_*loc*_ is the localization error, $\hat{P}$ and ***P***_*true*_ represent the estimated position and the true position respectively.

**Figure 3:**
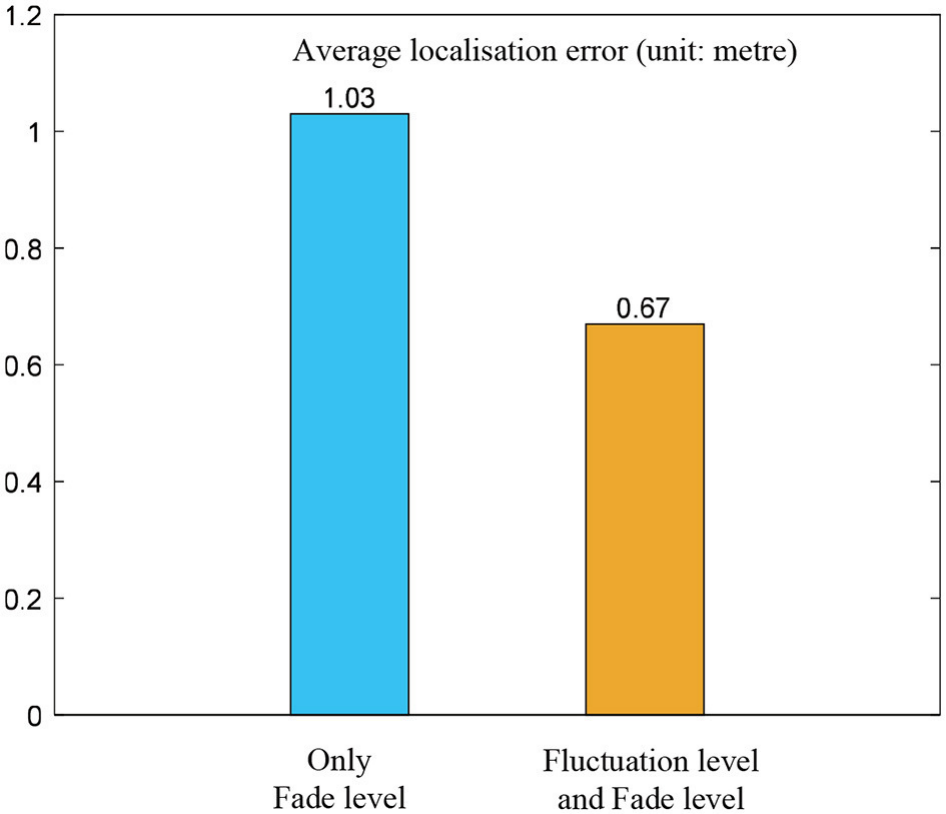
Comparison of localization error. Cyan represents the localization error only using fade level. Saffron yellow denotes the result when using both fluctuation level and fade level.

At each position, the localization error is computed following Equation (12). For being statistically effective, we compute the average localization error of all the positions as the metric for comparison. The average localization errors for two comparative strategies in selecting informative RSS (only fade-level vs. combining fluctuation-level and fade-level) are presented in Figure 3.

Obviously, the results in Figure 3 show that the strategy of combining fluctuation-level and fade-level can achieve lower localization error than that when just using fade-level.

## 4 Conclusion and discussion

For supervision of the patient to take home-based neuro-rehabilitation, this article explores the wireless device-free indoor localization technique to judge whether the patient is near the position of the rehabilitation-equipment. To reduce the localization error, this research proposes a new criterion (fluctuation-level) to combine with fade-level for the selection of informative RSS. Sparse Bayesian learning is used to reconstruct the shadowing image and estimate the position of the patient. Experimental results show that combining fluctuation-level and fade-level can achieve lower localization error than just using fade-level.

As to the mechanism, fluctuation-level shows the fluctuation property of the RSS measurements, this property is not reflected in fade-level. The combination of both can further filter the poor RSS that are ignored by using only fade-level. Future work will obtain the moving trajectory of the patient and build a rational formula from the amount of movement to the rehabilitation-efficacy, which is quite beneficial for neuro-rehabilitation evaluation.

## Data availability statement

The raw data supporting the conclusions of this article will be made available by the authors, without undue reservation.

## Ethics statement

The studies involving humans were approved by Ethics Committee of Shanghai University of Medicine & Health Sciences. The studies were conducted in accordance with the local legislation and institutional requirements. The participants provided their written informed consent to participate in this study.

## Author contributions

ZW: Conceptualization, Formal analysis, Methodology, Software, Writing – original draft. XL: Writing – review & editing. GW: Writing – review & editing.
